# A Treatise *on Inflammation*

**Published:** 1838-11-07

**Authors:** 


### BIBLIOGRAPHICAL NOTICE.

A TREATISE *on Inflammation.* By JAMES MACARTNEY, M. D., F. R. S., &c. &c. London, 1838. 4to. pp. 214.

THIS work contains the substance of the theory and practice on the subject of inflammation, taught by Dr. Macartney, in his lectures on surgery, in the University of Dublin. His views with regard to the connexion between inflammation and the restorative process, are original. According to his theory, this process does not depend upon inflammation, but is rather incompatible with it. Reunion and reorganization he supposes to be effected in four different ways:—

"*First*, by immediate union, without any intervening substance, such as blood or lymph.

"*Second*, the union by the medium of coagulable lymph, or a clot of blood.

"*Third*, re-organization without any medium of lymph or granulations, the cavity of the wound becoming obliterated by a natural process of growth.

"*Fourth*, the reparation by means of a new, vascular, and organized substance, called granulations.

"To the first of these modes of cure, I should wish to give the name of *immediate.* The second may be called the *mediate by lymph or blood.* The third, being compounded of different actions, I find a difficulty in distinguishing it by a single name. It might be denominated the *approximating* or the *modelling* process of reparation, or that by a *natural growth.* The fourth mode of union should be termed *mediate by granulation.*

"The three first mentioned modes of restoration are quite incompatible with the presence of inflammation; a slight degree of which may, however, exist with the fourth. Not that I admit the growth of granulations to be an inflammatory process in itself. It ought rather to be viewed as the mode of reparation, adopted under the unfavourable circumstances of irritation, or a degree of inflammation being still continued, and proves that parts previously in a healthy state, are disposed to heal in despite of many impediments thrown in their way.

"The circumstances under which immediate union is effected, are the cases of incised wounds, that admit of being, with safety and propriety, closely and immediately bound up. The blood, if any be shed on the surfaces of the wound, is thus pressed out, and the divided blood-vessels and nerves are brought into perfect contact, and union may take place in a few hours; and as no intermediate substance exists in a wound so healed, no mark or cicatrix is left behind." (p. 153–4.)

"The union of parts with the medium of lymph or blood takes place in wounds, which either cannot, from the extent or shape of their surfaces, be brought into perfectly close contact, or where the parts will not sustain much pressure, without the danger of inducing inflammation. The lymph which issues from the adjacent surfaces, in the first instance, glues them together, and in a few days is found to have acquired some vascularity, and an imperfect degree of organization; after which, an external restraint for keeping the divided surfaces in contact becomes unnecessary." (p. 155.)

The process of reparation by the modelling process, is thus described:—

"When healthy parts are injured, although it may be to the greatest extent, if placed under the most favourable circumstances for carrying on their natural actions, the process of reparation is nearly the same, even in the human subject, as that which I have described as belonging to the animals of a simple structure. The pain arising from the injury soon ceases. No tumefaction ensues separating the edges of the wound, and its surfaces are not only disposed to lie in contact, but even to approach each other so much that they cannot be kept asunder by mechanical restraint; there is, therefore, no necessity for the effusion of lymph; and as there is no cavity to be filled up, granulations are not formed. The surfaces of the wound, although they come into contact, do not unite by vessels shooting across; they are smooth, red, and moistened with a fluid, which is probably serum, and present the appearance of one of the natural mucous surfaces of the body. If any parts have been killed by the injury, they are separated by simply as much interstitial absorption as is sufficient to set them free. The wound is finally healed by the same means which determine the shape of the natural parts of the body. It gradually diminishes in extent until it is obliterated, or it may be cicatrized before the surfaces are abolished; after which the same process of natural growth goes on, until no part of the original wound is left. The cicatrix which succeeds the cure of the injury by the modelling process, is small, pliant, free from those callous adhesions to the parts underneath, and the morbid sensations that so often belong to those cicatrices which have for their bases the deposits of lymph or the formed structures called granulations. When the modelling process or cure by natural growth goes on perfectly, there is no inflammation in the part, and the patients are so entirely free from all uneasy sensations, that I have known instances of their being ignorant of the real site and extent of the injury, until they had examined the part with their hand, or saw it in a looking-glass." (p. 53,—4.)

Dr. Macartney's chapter on the remedies for inflammation, contains some instructive suggestions:—

*Tartar emetic* he uses in smaller than the usual doses: "the good effects of nausea depend on the feeling being steadily kept up for some time, and this can best be accomplished by very divided and frequently repeated doses of the medicine." Externally, the remedial operation of a *moderate degree* of cold, is, he thinks, in most cases to be preferred. The application of *intense cold* is reserved for very severe injuries, compound luxations, for example, where the inflammation cannot be restrained by other means. The operation of cold and moisture must be uninterrupted to be of use. Irrigation, by means of the syphon, effects this purpose. The alternations of heat and cold to an inflamed part, which result from the imperfect renewal of cloths, dipped in refrigerant lotions, are necessarily hurtful; and the plan of irrigation, which we owe to the French, is a valuable improvement in surgery. The importance of the steady employment of external applications, Dr. Macartney illustrates by his success with lead water in *tinea capitis*. He says that—

"The lead lotion never fails to cure tinea capitis, however long and obstinately the complaint may have resisted other remedies, provided the application of the lotion be properly conducted. The hair should be first shaved off; water dressing, or a poultice of any kind, is then to be applied, merely for the purpose of cleansing the skin of the crust, and all other impurities. There will then be seen, under each crust, a red spot of the skin, denuded of its cuticle, and the villous surface exposed. The lotion should now be applied by means of lint, thoroughly wetted with the fluid, and covered with a plate of indian rubber, or a piece of oiled silk to prevent evaporation. Every time this dressing is changed, which should be very frequently at first, the head should be washed with some of the lotion, and the lint should be replaced by some that is clean, which is to be completely wetted with the lotion, and covered as before. The efficacy of this mode of treatment depends entirely on the head being continually subjected to the operation of an astringent fluid; for, if the application be suspended for one night, or even for a few hours, the peculiar viscid secretion which forms the crusts will re-appear, and the whole treatment will have to commence again." (p. 172,—3.)

Remedies which *affect in an agreeable manner* the sensations of inflamed parts, according to Dr. Macartney, have a powerful tendency to subdue inflammation. The application of *steam*, at a suitable temperature, to the skin, he has found the best means we possess of producing a grateful state of sensation. He has invented an apparatus for the employment of steam, from the hottest degree at which it can be borne, down to below the standard temperature of the human body. We give his description of it at length.

"It consists of a small tin boiler, supported on a platform, on which a spirit lamp is placed. The peculiarity of the vessel is, that the superior opening is an expanded funnel, in consequence of which the steam ascends from the boiler with a vertical motion, being attracted to the smooth and infundibular aperture. The effect of the steam escaping in this manner is to diffuse and cool it so much, that if the vessel be uncovered, the hand may be placed within an inch of the surface of the boiling water, without experiencing any unpleasant feeling of heat, although, if the steam be made to spread in a straight line, by diminishing the opening of the funnel, it will scald the hand held two or three feet above the water. The steam is conducted to any part of the body, by means of a tube of woollen cloth, about twelve inches wide and three feet long. Its cylindric form is maintained by circular pieces of whalebone. One end of the woollen tube is tied round the contracted neck of the boiler, and the other end admits of being adapted to the shape of any part that is intended to be fomented, from holding within its opening a piece of flexible wire.

"By this apparatus, steam at any temperature may be applied for any length of time, with only the momentary interruption of renewing the boiling water, and the spirit in the lamp. The great advantage of making a continued, instead of a temporary application, at a determined temperature, and without the intervention of the woollen cloths used in common fomentation, which irritate many wounds and ulcers, gives to the mode I have described for administering steam, the character of a *new remedy*, which it exhibits also in the more extended and more beneficial effects than those of common fomentation."

"The effects of steam, at a high but comfortable temperature, are gently stimulant, and extremely soothing to the feelings of the patient. When used immediately after the receipt of lacerated gunshot and punctured wounds, contusions of bones, fractures near joints, recent luxations, bruises and strains of joints, and in all wounds accompanied with a peculiar overcoming pain, and a shock to the nervous system, it removes all pain, and consciousness of injury in a short time. After the pain and sense of injury have passed away, the steam may be continued at a lower temperature. Hot steam is remarkably successful in improving the condition of the indolent ulcer; and for inflammation of a more active character, no local application can compete with steam at a low temperature." (p. 176,—7,—8,—9.)

The *water dressing* consists of two or three layers of lint, floated in the water before being folded, and covered with French oiled silk or indian rubber, which should project beyond the margin of the lint, to prevent evaporation. It is to be changed two or three times a day, and is recommended as having not only better, but very different effects from poultices. It presents or diminishes the secretion of pus, and under its use, granulations, which are rendered exuberant by poultice, are either never formed or exist in a slight degree. Very ancient authority is cited for the employment of water as a remedy for wounds and inflammation, but Dr. Macartney claims the credit of having introduced it to the attention of the profession in modern times. For this and other valuable practical suggestions, he is entitled to our thanks; and, as far as we know, the novelty of his views, as to the possibility of open wounds healing without inflammation, and without the medium of either coagulable lymph or granulation, cannot be disputed.

